# Asymptomatic *Cryptosporidium* infections in ewes and lambs are a source of environmental contamination with zoonotic genotypes of *Cryptosporidium parvum*

**DOI:** 10.1051/parasite/2020054

**Published:** 2020-11-03

**Authors:** Léa Bordes, Pauline Houert, Damien Costa, Loïc Favennec, Corinne Vial-Novella, Francis Fidelle, Christelle Grisez, Françoise Prévot, Philippe Jacquiet, Romy Razakandrainibe

**Affiliations:** 1 IHAP, UMT Pilotage de la Santé des Ruminants, Université de Toulouse, INRAE, ENVT 31076 Toulouse France; 2 Centre Hospitalier Universitaire, Centre National de Référence – Laboratoire Expert des Cryptosporidioses 76031 Rouen France; 3 Centre Départemental pour l’Elevage Ovin, Quartier Ahetzia 64130 Ordiarp France

**Keywords:** *Cryptosporidium parvum*, Lamb, Ewe, Zoonotic genotypes, PCR, Asymptomatic infection

## Abstract

Protozoan parasites of the *Cryptosporidium* genus cause severe cryptosporidiosis in newborn lambs. However, asymptomatic infections also occur frequently in lambs and ewes. In sheep, the most commonly detected *Cryptosporidium* species are *C. ubiquitum*, *C. xiaoi* and *C. parvum.* Due to a lack of relevant information about such infections in France, we investigated the situation on five dairy sheep farms in the Pyrénées-Atlantiques Department in south-western France in December 2017. Individual fecal samples were collected from 79 female lambs (5–17 days old) and their mothers (72 ewes). Oocysts were screened using Heine staining before and after Bailenger concentrations. *Cryptosporidium* species identification and genotyping were performed using real-time PCR and *gp60* gene sequencing. No cases of clinical cryptosporidiosis were observed in the 79 lambs. Microscopically, *Cryptosporidium* spp. oocysts were observed in only one lamb on one farm (prevalence 1.3%) and one ewe on another farm (prevalence 1.4%). By contrast, *Cryptosporidium* spp. DNA was detected in 17 ewes (prevalence ranging from 10.5% to 50% depending on the farm) and in 36 lambs (prevalence ranging from 0% to 77.8% depending on the farm). Only zoonotic *Cryptosporidium parvum* IId and IIa genotypes were identified when genotyping was possible. *Cryptosporidium ubiquitum* and *C. xiaoi* were detected on one and three farms, respectively. We conclude that healthy young lambs and their mothers during the peripartum period could be a source of environmental contamination with oocysts.

## Abstract (fr)

Les parasites protozoaires du genre *Cryptosporidium* provoquent une cryptosporidiose sévère chez les agneaux nouveau-nés. Cependant, des infections asymptomatiques surviennent aussi fréquemment chez les agneaux et les brebis. Chez les ovins, les espèces de *Cryptosporidium* les plus couramment détectées sont *C. ubiquitum*, *C. xiaoi* et *C. parvum*. En raison d’un manque d’informations pertinentes sur ces infections en France, nous avons enquêté sur la situation de cinq élevages ovins laitiers des Pyrénées-Atlantiques en décembre 2017. Des échantillons fécaux individuels ont été collectés sur 79 agnelles (5 à 17 jours) et leurs mères (72 brebis). Les oocystes ont été criblés en utilisant une coloration Heine avant et après concentration par la technique de Bailenger. L’identification et le génotypage des espèces de *Cryptosporidium* ont été réalisés à l’aide de la PCR en temps réel et du séquençage du gène *gp60*. Aucun cas de cryptosporidiose clinique n’a été observé chez les 79 agneaux. Au microscope, les oocystes de *Cryptosporidium* spp. n’ont été observés que chez un agneau dans une ferme (prévalence 1,3 %) et chez une brebis dans une autre ferme (prévalence 1,4 %). En revanche, de l’ADN de *Cryptosporidium* spp. a été détecté chez 17 brebis (prévalence allant de 10,5 % à 50 % selon les fermes) et chez 36 agneaux (prévalence variant de 0 % à 77,8 % selon les fermes). Seuls les génotypes zoonotiques de *Cryptosporidium parvum* IId et IIa ont été identifiés lorsque le génotypage était possible. *Cryptosporidium ubiquitum* et *C. xiaoi* ont été détectés respectivement dans une et trois fermes. Nous concluons que les jeunes agneaux en bonne santé et leurs mères, autour de l’agnelage, pourraient être une source de contamination environnementale par les oocystes.

## Introduction

*Cryptosporidium* spp. are ubiquitous protozoan parasites, responsible for the gastrointestinal disease cryptosporidiosis. Species of *Cryptosporidium* can infect a wide range of vertebrate hosts, including humans. *Cryptosporidium* infection is one of the leading causes of diarrhea morbidity and mortality in children younger than five years and is associated with severe life-threatening illness among immunocompromized patients [18, 22, 45]. *Cryptosporidium* oocysts shed with the feces of a host are immediately infective. Infection with *Cryptosporidium* oocysts can be acquired through (i) the fecal-oral route, (ii) contaminated water or food, or (iii) aerosolized droplets or by contact with fomites contaminated by coughing [35]. Some species, such as *Cryptosporidium parvum*, appear to lack host specificity as they can be found in a wide range of hosts. In fact, the latter species display considerable adaptation abilities due to their high genetic variability [12]. Cryptosporidiosis has become a public health and veterinary concern as livestock can act as a reservoir and source of zoonotic cryptosporidiosis [53]. Livestock, young calves, lambs and goat kids are highly susceptible to the parasite and can develop severe diarrhea with high mortality rates, causing significant economic losses associated with anorexia, impaired growth, and death of the animal [41]. The infection can spread on the farm via the fecal-oral route by environmental contamination and animal interactions, such as during suckling [32, 52, 54]. The major sources of contamination for humans are drinking and recreational waters contaminated by livestock [11] or infected humans. However, few clinical human cryptosporidiosis cases can be explained by environmental contamination with sheep and goat manure due to the difficulty of tracking down the initial source of contamination [38]. In their model, Vermeulen and colleagues [49] estimated the *Cryptosporidium* oocyst loads in livestock manure and predicted that it was the main source of environmental contamination in Europe and North America. Without adequate control, this contamination represents a human health hazard, because animals infected with *C. parvum* could be excreting up to 10^7^ oocysts per gram of feces [11].

**Table 1:** Prevalence of *Cryptosporidium* infections in lambs and ewes determined by molecular analysis.

Farms	Animals	Number examined	Animals positive for *Cryptosporidium* species		*Cryptosporidium parvum* genotyping
			*18S rRNA* gene-based PCR	*Lib13* locus and *18S rRNA* sequence analysis	
#1	Lambs	20	13 (65%)	13 ND	
	Ewes	18	3 (16.7%)	2 *C. parvum* *	1 *C. parvum* IIdA21G2
				1 ND	1 *C. parvum* IIdA15G1
#2	Lambs	8	4 (50%)	1 *C. xiaoi*	
				3 ND	
	Ewes	9	3 (33.3%)	1 *C. parvum* *	1 ND
				1 *C. ubiquitum*	
				1 ND	
#3	Lambs	18	14 (77.8%)	2 *C. parvum* *	2 *C. parvum* IIdA24G1
				12 ND	
	Ewes	10	5 (50%)	1 *C. xiaoi*	
				4 ND	
#4	Lambs	13	0		
	Ewes	16	2 (12.5%)	1 *C. parvum* *	1 *C. parvum* IIaA16G3R1
				1 ND	
#5	Lambs	20	5 (25%)	2 *C. parvum* *	2 ND
				3 ND	
	Ewes	19	2 (10.5%)	2 *C. parvum* *	1 *C. parvum* IIaA16G3R1
					1 *C. parvum* IIaA13G2R1
Total	Lambs	79	36 (45.6%)	4 *C. parvum* *	2 *C. parvum* IIdA24G1
				1 *C. xiaoi*	
				31 ND	
	Ewes	72	17 (23.6%)	6 *C. parvum* *	2 *C. parvum* IIaA16G3R1
				3 *C. xiaoi*	1 *C. parvum* IIdA21G2
				1 *C. ubiquitum*	1 *C. parvum* IIdA15G1
				7 ND	1 *C. parvum* IIaA13G2R1

ND: not determined due to sequencing failure.

* Determined by Lib13 PCR result.

Clinical infections have been extensively investigated, especially in calves [3, 44, 47]; however, the prevalence of asymptomatic parasite infections and their consequences are less well documented. A few studies have investigated the prevalence of asymptomatic *Cryptosporidium* spp. infections in calves, sheep and goats [33, 38, 40, 52], and underlined the importance of identifying the species and genotypes involved to control zoonotic disease and environmental contamination better.

To assess the prevalence of *Cryptosporidium* spp. infections in humans or animals, fecal samples are tested. Various methods can be used, such as microscopic staining methods or immunofluorescence detection. These approaches are easy to use and require only basic laboratory equipment but are less sensitive [5] compared to molecular methods. They also require technical expertise, for a result that remains subjective. Molecular methods were developed to increase the sensitivity and specificity of diagnosis and to provide species-level information and the genotype of the parasite. PCR assays, targeting the 18S rRNA gene, are used for species-level identification [21]. To provide further information, molecular genotyping techniques have been developed. It is useful to identify genotypes of potential anthropozoonotic and zoonotic transmission.

In France, many studies have investigated *Cryptosporidium* spp. infections in livestock, especially in goat kids [4, 8, 35] and in calves [9, 23, 38, 39]. Still, scarce data are currently available in the literature regarding *Cryptosporidium* infections in lambs and sheep.

The aim of this study was, therefore, to investigate the prevalence and zoonotic potential of asymptomatic *Cryptosporidium* spp. infections in postpartum ewes and neonatal lambs on farms in the Pyrénées-Atlantiques Department of south-western France.

## Materials and methods

### Ethics statement

Before carrying out this work, informed written authorization to perform and to publish the present epidemiological study anonymously was obtained from all owners. Stool collection is a part of routine veterinary procedures without any traumatic method. Such procedures are not qualified as animal experimentation involving vertebrates according to French laws, and no specific ethical clearance was required.

### Fecal samples from lambs and ewes

In December 2017, the presence of *Cryptosporidium* spp. was investigated on five volunteer farms rearing Blond-Faced Manech dairy sheep in the French Basque Country, part of the Pyrénées-Atlantiques Department. All were mixed farms with a dairy sheep flock and a beef cattle herd. On the five farms studied, grouped artificial inseminations were performed to have only one lambing period per year. Ewes came inside a month before lambing and remained indoors until lambs were weaned at 1 and a half months. Regarding breeding practices, lambs on farm #3 were separated from their mothers immediately after receiving colostrum and then fed artificially. On the contrary, animal management practices were apparently identical within farms #1, #2, #4 and #5: lambs were born in the shed and stayed with their mother until they were 1.5 months old, before being separated from them. On all farms, lambs remained indoors during this study. In total, 79 female lambs, aged from 5 to 17 days and their mothers (72 ewes) were tested during the study. From each farm, 8–20 female lambs and 9–19 ewes were analyzed (Table 1). For each animal, an individual fecal sample was obtained by rectal stimulation, and the clinical status was evaluated and recorded, especially signs of diarrhea. Fecal samples were stored at 4 °C before microscopic and molecular analysis.

### Microscopic detection of *Cryptosporidium* spp. oocysts in fecal samples

The presence of *Cryptosporidium* spp. in fecal samples was determined using Heine staining by direct examination [34] and after Bailenger concentration [1]. Briefly, for the negative staining technique of Heine, 10 μL of fecal matter with no preservative were mixed with an equal amount of undiluted carbol-fuchsine solution on a microscope slide. A thin smear was prepared, allowed to air dry and examined within 15 min under phase-contrast microscope, a hundred microscopic fields were observed using an oil-immersion objective of ×40 magnification. *Cryptosporidium* oocysts appear as unstained, strongly refractive, round to oval structures of about 3–6 μm in diameter. The presence of other intestinal parasites (*Giardia*, *Strongyloides*, and amoeba) detected in some of the ewe and lamb fecal samples was not considered in the present study.

### Cryptosporidium species identification and genotyping

All samples were analyzed by molecular methods for species identification, and positive samples were genotyped in a second step. Samples were prepared as described by Razakandrainibe and colleagues [38]. Briefly, 250 mg of feces were pre-treated using mechanical lysis in Lysing Matrix A Tubes (Qiagen, CA, USA), thermal shock lysis and sonication before isolating DNA from the pre-treated samples using a modified QIAmp Stool Mini Kit (Qiagen, CA, USA).

*Cryptosporidium* species were screened using real-time PCR targeting the *18S rRNA* and *LIB13* genes, as described by Hadfield and colleagues [17]. Briefly, PCR was carried out in duplicate and consisted of two duplex reactions: (i) a genus-specific PCR amplifying ∼300 bp of the *Cryptosporidium 18s rRNA* gene, duplexed with a *C. parvum*-specific PCR amplifying 166 bp of the *LIB13* locus, and (ii) a *C. hominis*-specific PCR amplifying 169 bp of the *LIB13* locus.

Thermocycling conditions were as follows: 95 °C for 10 min, followed by 55 cycles of 95 °C for 15 s and 60 °C for 60 s. Data were collected from each probe channel during each 60 °C annealing/extension phase.

Samples positive with the species-specific probes were then further characterized by examining the *gp60* gene. However, as the primers commonly used for *gp60* subtyping of *C. parvum* and *C. hominis* do not reliably amplify many other *Cryptosporidium* species, DNA samples that only reacted with the genus-specific probes were also sequenced at the *18S rRNA* gene for species identification.

Genotyping was performed by sequencing a fragment of the *gp60* gene. Primers AL3531 and AL3533 were used in the primary PCR and primers AL3532 and LX0029 in the secondary PCR, leading to amplification of a fragment of approximately 364 bp [16]. Each PCR mixture (total volume, 50 μL) contained 5 μL of 10X DreamTaq Buffer, each deoxynucleoside triphosphate at a concentration of 0.2 mM, each primer at a concentration of 100 nM, 2.5 U of DreamTaq polymerase, and five microliters of DNA template. Also, 1.25 μL of DMSO (100%) were added to the mixture. A total of 40 cycles, each consisting of 94 °C for 45 s, 55 °C for 45 s, and 72 °C for 1 min, were performed. An initial hot start at 94 °C for 3 min and a final extension step at 72 °C for 7 min were also included. Each amplification run included a negative control (PCR water) and two positive controls (genomic DNA from purified *C. parvum* oocysts (from experimentally infected calves) purchased from Waterborne Inc., and *C. hominis* genomic DNA from a fecal specimen collected in Rouen University Hospital). Products were visualized in 2% agarose gels using ethidium bromide staining. Positive samples were further genotyped by DNA sequencing of the *gp60* gene.

Sequencing was used to confirm *Cryptosporidium* species/genotypes from second-round PCR products. PCR amplicons were purified using Exonuclease I/Shrimp Alkaline Phosphatase (Exo-SAP-IT) (USB Corporation, OH, USA), and were sequenced in both directions using the same PCR primers at 3.2 μM in 10 μL reactions, Big Dye™ chemistries, in an ABI 3500 sequence analyzer (Applied Biosystems, CA, USA). Sequence chromatograms of each strand were examined with 4 peaks software and compared with published sequences in the GenBank database using BLAST (https://www.ncbi.nlm.nih.gov/BLAST). Genotypes were named using the established *gp60* genotype nomenclature [46]. The sequences obtained in this study were deposited in GenBank under the accession numbers MT418843–MT418848. In case of failure in species/genotype identification, ND (not determined) was annotated to the corresponding isolate in Table 1.

### Statistical analysis

Statistical analyses were performed with R software, version 3.6.3 (2020/02/29) using R studio, version 12.5033. A Fisher’s exact test was used to compare the prevalence of cryptosporidiosis between farms and animal categories.

## Results

Microscopically, results showed a low prevalence of animals infected with the parasite: oocysts were detected in only one lamb (1.3%) on farm #5 and one ewe (1.4%) on farm #2. Molecular characterization showed that *C. ubiquitum* infected the lamb; however, identification of the *Cryptosporidium* species infecting the ewe was unsuccessful due to an unreadable sequence (peaks unevenly spaced, nucleotide bases were not deciphered correctly). At the farm level, the prevalence ranged between 0% and 5% for lambs, and between 0% and 11.1% for ewes. During this study, no clinical cases of cryptosporidiosis were observed.

The results of the PCR analyses (species identification and genotyping) are provided in Table 1. Out of the five investigated farms, four were found to have asymptomatic cryptosporidiosis in lambs: only farm #4 was free of *Cryptosporidium* DNA in lamb feces. In ewes, *Cryptosporidium* DNA was detected on all of the studied farms. The prevalence of *Cryptosporidium* infection was higher with PCR analysis than with microscopic examination of feces, regardless the farm considered. The 18S rRNA real-time PCR revealed asymptomatic infection in 45.6% and 23.6% in lambs and ewes, respectively. At the farm level, the prevalence ranged from 0% to 77.8% in lambs with significant differences between farms (*p*-value = 1.138e-5), where farms #1, #2 and #3 prevalence values are significantly higher than farms #4 and #5, and from 10.5% to 50% in ewes with no significant differences between farms (*p*-value = 0.1021). Many animals found to be negative using microscopy had positive results with DNA detection (data not shown). The prevalence of asymptomatic *Cryptosporidium* spp. infection was higher in lambs than in ewes (except on farm #4, where no *Cryptosporidium* infections were detected in lambs). High between-farm variability was observed for the prevalence. Farm #3 had the highest prevalence of infection in lambs (77.8%) and ewes (50%).

The 18S rRNA sequence analysis from readable electrophoregrams revealed three species: *C. parvum*, *C. xiaoi* and *C. ubiquitum*. For the rest, analysis showed multiple overlapping traces after a point in the sequence, low/poor signal-to-noise ratio in sequence data, and premature termination of sequences causing unreadable sequences. *Cryptosporidium parvum* was detected on all farms, *C. xiaoi* on three farms (farms #1, #2, and #3), and *C. ubiquitum* only on farm #2. More precisely, *C. parvum* and *C. xiaoi* were identified in both lambs and ewes, whereas *C. ubiquitum* was detected only in ewes. In lambs, *C. parvum* was detected on farms #3 and #5 (2/14 lambs and 2/5 lambs, respectively) and *C. xiaoi* on farm #2 (1/4 lambs). In ewes, *C. parvum* was identified on farms #1, #2, #4, and #5, *C. xiaoi* was detected on both farms #1 and #3, and *C. ubiquitum* was only found on farm #2.

The Lib13 PCR specifically amplified 10 *C. parvum* of which three led to *gp60* unreadable superimposition of electrophoregrams. *Cryptosporidium hominis* were not found in this study. Two *C. parvum gp60* genotype families were identified: IId and IIa. More precisely, three different IId genotypes were detected, IIdA24G1, IIdA21G2, IIdA15G1, as well as two different IIa genotypes, IIaA16G3R1 and IIaA13G2R1. The *gp60* IIdA24G1 genotype was detected in lambs on farm #3 but could not be detected in ewes, if present, due to a failure on *gp60* genotyping on this farm.

## Discussion

This study described prevalence of asymptomatic *Cryptosporidium* infections in lambs and ewes on five farms in the Pyrénées-Atlantiques Department in France. Our study is subject to certain limitations that should be considered when interpreting the results. We recognize a major key limitation of the study is the discrepancies between the number of positive samples detected with the 18S rRNA PCR assay and the sequence-based validated isolates. Determining *Cryptosporidium* species has been impeded by technical limitations. Mixed *Cryptosporidium* species could explain the sequencing difficulties encountered in this work. The simultaneous presence of several species in the same sample could lead to amplification and sequencing of different genetic fragments, leading to unreadable superimposition of electrophoregrams. However, it is noteworthy that this prospective cohort study highlighted, when genotyping could be performed, frequent characterization of *C. parvum* with zoonotic genotypes. *Cryptosporidium parvum* infections are common in Europe [28, 31, 37, 42], but not on other continents, where *C. xiaoi* and *C. ubiquitum* predominate [42].

In this present study, despite the high proportion of positive cases detected using the *18S rRNA* gene (rDNA)-based primers, sequence analysis showed low signal-to-noise ratios, overlying sequences, and premature termination of sequences, and application of this technique to routine analysis seems complicated. Other studies, using the selected primers, pinpointed sequence homologies with the yeast *18S rRNA* gene (GenBank accession number n° JN940588.1) [26]. One potential source of bias in our study is detection bias as sample collection was carried out at a single point in time. Adult sheep are known to excrete *Cryptosporidium* oocysts, but the infection is always asymptomatic with a low level of excretion [30]. Oocyst excretion is known to increase in ewes during parturition and represents a significant source of contamination both for the environment and their lambs [30, 52]. All the ewes sampled belonged to the first wave of lambing, so their lambs were not in contact with other older lambs. There were, therefore, only two potential sources of contamination for newborn lambs: (i) *Cryptosporidium* oocysts excreted by the ewes, and (ii) residual contamination of the environment by oocysts from the previous lambing season. The sampling dates, the age of lambs sampled, and the breed (Blond-Faced Manech) were similar; however, the results were highly variable from farm to farm.

Regarding animal management practices, farm #3 fed lambs artificially. On this farm, lambs, once separated from their mothers, are grouped together to be fed artificially. The density of young and naïve animals is high, which could explain the higher prevalence here than on other farms in lambs, but not in ewes. The other farms have lower prevalence in lambs and ewes with their breeding practices, and the lambs from farm #4 were not infected with *Cryptosporidium* spp., with the prevalence of infection being very low in their mothers (12.5%).

Three *Cryptosporidium* species were identified in this study: *C. parvum* was frequently detected, *C. xiaoi* and *C. ubiquitum* were also identified. The latter two species have already been reported in human cryptosporidiosis cases, although the zoonotic potential is certainly much lower than that of *C. parvum* [23].

These species are commonly found in sheep and goats [11, 37, 43]. *Cryptosporidium andersoni*, which is known to infect sheep [12], was not detected in the present study. Likewise, *C. hominis* was detected neither in lambs nor in ewes. In rare cases, it can infect goats and sheep [15] but is found with a higher prevalence in calves [38]. As the studied farms were mixed farms with both dairy sheep and meat cattle, we had expected to detect *C. hominis,* but this was not the case. However, *Cryptosporidium* spp. infections in calves were not investigated during this study.

Regarding *C. parvum* genotypes, two different zoonotic genotype families of *gp60* IIa and IId were detected. The presence of these genotypes is well described in sheep and goats [2, 12, 37, 48].

Genotype IIdA24G1 was found only in lambs, consistent with studies performed in north-eastern Europe [19, 37]. In 2010, this genotype caused foodborne outbreaks of cryptosporidiosis in Sweden [14]. The other IId genotype, IIdA15G1, was reported to be common in calves in China [7, 36] but not in ewes as in this study. One human case of infection has been reported in a Slovak patient [27], and in cattle and humans in both Iran [29] and the Netherlands [51]. The last IId genotype, IIdA21G2, was found only in ewes in this study. To our knowledge, it has not been reported in the literature, indicating that it could be a new genotype infecting sheep.

Two IIa genotypes were found in ewes: IIaA13G2R1 and IIaA16G3R1. The first genotype has been described in lambs and goat kids in Algeria [2] and calves in Belgium [13]. It was also found in a patient with acute diarrhea in South Korea [24] and in patients from Malaysia with HIV/AIDS [20]. The second one has been reported in lambs and kids with clinical cryptosporidiosis in France [25] and in Spain [10], and more surprisingly in asymptomatic infections in wild ponies of the Iberian Peninsula [6]. This genotype has been found in children in Iran [29] and in sporadic infections in Canada [47] and Australia [50].

Some samples could not be defined at the genotype level due to: (i) insufficient amounts of DNA to have satisfactory quality of genotyping; and (ii) the presence of multiple genotypes of *C. parvum* in the sample, resulting in an illegible electrophoregram. This last point may also be due to mixed co-infections of several genotypes of *Cryptosporidium parvum* in the sample.

Each farm has a specific association of *Cryptosporidium* species and genotypes, although the breeding conditions were very similar. On the same farm, infections in lambs and ewes were not identical, some species or genotypes being found in one category of animals only. Several reasons could explain this difference. The first is an underestimation of the number of different *Cryptosporidium* spp. present in lambs. Clearly, since infections are asymptomatic, only a limited number of oocysts are excreted in feces [30], and the amount of extracted DNA could be insufficient for molecular analysis. In many cases, therefore, the parasites may be present, but the species and genotype cannot be detected.

Furthermore, only a portion of the herd on each farm was sampled and analyzed individually in this study. The results, therefore, reflect the *Cryptosporidium* species and the genotypes found in a fraction of the animals present on a farm.

The comparison of *Cryptosporidium* species and genotypes present in ewes and their lambs does not seem in favor of contamination of the lamb only by its mother. Due to the fecal-oral transmission mode of the parasite and its resistance in the environment, lambs can be infected by all *Cryptosporidium* oocysts excreted by all ewes and present in the environment. Therefore, our sampling effort was probably not sufficient to provide a complete description of *Cryptosporidium* associations and to better understand the natural transmission of the parasite from ewes to lambs.

## Conclusions

This study investigated *Cryptosporidium* infections in lambs and ewes on dairy sheep farms in the Pyrénées-Atlantiques, France. Molecular analysis revealed asymptomatic infection by *Cryptosporidium* spp. The three *Cryptosporidium* species identified were *C. parvum*, *C. xiaoi*, and *C. ubiquitum*. In the present study, zoonotic *Cryptosporidium parvum* IIa and IId genotypes were detected and may highlight the possible role of lambs and ewes as a source of infection, and a potential zoonotic reservoir for human infections. Considering the low number of animals and farms investigated in this study, it would be interesting to confirm these data on other farms. Multiyear monitoring of the same herd could also provide useful information about the evolution of species and genotypes over time.

## Conflicts of interest

The authors declare that there were no conflicts of interest at any point in time.
